# Preference for Street Environment Based on Route Choice Behavior While Walking

**DOI:** 10.3389/fpubh.2022.880251

**Published:** 2022-08-05

**Authors:** Lan Jin, Wei Lu, Peijin Sun

**Affiliations:** School of Architecture and Fine Art, Dalian University of Technology (DUT), Dalian, China

**Keywords:** walking behavior, street environment, walking preference, route choice characteristics, the shortest distance route

## Abstract

This study aimed to better understand the relationship between the street environment and walking behavior by deciphering the pedestrians' street environment preference based on their route choice behavior while walking. The route data of 219 residents were collected using an unobtrusive tracking method and subjected to binary logistic regression models to analyze the pedestrian route choice behavior. The results revealed that except for the walking distance, the trip purpose and travel status are the potential factors influencing the route choice of pedestrians. Furthermore, it was revealed that on-street parking, garbage bins, and streetlights could influence the pedestrians to select longer distance routes. In addition, pedestrians were more likely to select the shortest distance route when they were engaged in leisure activities with an accompanist. The findings of this study would offer insights, from different perspectives, into the micro-scale street environment and the walking behavior of pedestrians.

## Introduction

Walking is the simplest mode of transportation that may be incorporated as a first-mile or last-mile solution into a wider transportation network, ultimately reducing the negative impacts of automobiles on the environment (1, 2). In addition, walking is one of the most widely used forms of physical activity that assists in preventing various physiological and mental disorders associated with a sedentary lifestyle, such as obesity, diabetes, and depression (3–5). Therefore, improving the environment of pedestrians within a community is beneficial in several ways.

The simplest and the most acceptable form of any physical activity for most people is one that may be integrated into their daily lives (6). Utilitarian walking is a type of walking that refers to undertaking walking trips to routine destinations, such as shops, school, work, and bus stops. This kind of walking has been identified as a core factor that supports a sustainable lifestyle and allows achieving the recommended levels of positive health outcomes of the World Health Organization (WHO) with just 10 min of daily bouts of physical activity (2, 7). However, the COVID-19 pandemic is causing mass disruption to our daily life, especially the dramatic declines in utilitarian walking, which results in the impact of the economic benefits of the area (8, 9). Therefore, enhancing the frequency and duration of utilitarian walking is considered an effective method for improving one's physical activity.

The relationship between the built environment and walking has been a research hotspot in several fields of science, such as transportation, urban planning, and public health. Several scholars believe that community design and land use development affect the choice of the mode of transportation (walking behavior). Numerous studies, ranging from macro- to micro-scales, have attempted to understand the relationship between the built environment and the walking behavior of people (10, 11). The macro-scale studies have been more focused on the land-use factors, such as density, diversity, design, distance, and destination (3Ds or 5Ds), which are, in turn, connected to walking behavior, such as walking distance, frequency, and choice of the mode of walking (12–14). On the other hand, the micro-scale studies are based on the relationship between the quality of the street environment and the walking behavior, including the quantity and quality of the pedestrian infrastructure, such as sidewalks, their width, their condition, and their separation from the traffic, along with the likelihood of the walking trips (15, 16). The other factors important in the context of walking include certain amenities, such as parks and open spaces, streetscape greenery, and urban design qualities such as imageability, enclosure, human scale, transparency, and complexity (17–19).

Most studies aimed at measuring the different aspects of walking behavior rely on area-based measurements, and the survey data are usually collected at the census collector district level in spatial analysis units. Such studies have mostly relied on comparing the differences in the walking amounts under different environmental characteristics (20). However, a major challenge encountered in the empirical evaluation of the relationship between the built environment and walking behavior is residential self-selection, which is caused by the inability to conduct intervention trials on the built environment within the community (21). For instance, people who prefer to walk select a neighborhood with a pedestrian-friendly environment to reside in, which leads to a bias in the estimated relationship between the built environment and walking behavior without exogenous variations in the sample residential location (21). Although various strategies have been adopted to address this residential self-selection, residents in different neighborhoods often belong to different social groups and their personal differences, such as those in attitudes and preferences for travel, remain unobserved in empirical research (21, 22). In addition, aggregated, rather than disaggregated, approaches are usually adopted when studying the relationship between the built environment and walking behavior (20). For instance, the concept of travel behavior includes travel distance and daily travel frequency and duration. This concern is further evident in public health research, where the walking data are in the form of frequency, intensity, and duration, while the analysis of the built environment variables is rare (20). Therefore, it is imperative to identify a specific measure for evaluating and predicting the impacts of environmental modifications on actual walking.

Route choice behavior refers to the decision-making process employed when pedestrians select one route among all possible routes connecting two consecutive destinations. The route choice reflects the decision-making behavior adopted during the walking process after determining the travel mode and the output result based on the interaction between the linear-segment street environment and the adopted walking behavior. Therefore, analyzing the route choice behavior would enable overcoming the walking behavior measurement method-related limitations of the traditional research on the street environment and walking behavior. Specifically, compared to area-based measures, route-based measurements better reflect the pedestrians' walking experience of the environment during the walking process or enable capturing the slight differences in the personal preferences for the street environment (23). Therefore, depending on trip assignment in the field of travel behavior, route choice enables a specific understanding of the pedestrians' preferences for the environment while Moreover, the biggest advantage of route choice is that it is less likely to correlate with residential location decisions which may partially overcome the concerns of self-selection. Also, most of the studies conducted on route choice compare the selected and non-selected routes and provide valuable insights into the environment-behavior interactions and route choice considerations. This type of comparison is also intuitively appealing, as routes serve as the ways through which pedestrians' would experience and relate to the street environment (21, 23). Therefore, understanding the route choice behavior of pedestrians would enable analyzing and deciphering the relationship between the street environment and walking behavior from different perspectives.

The objective of this study was to reveal the pedestrians' preference for the street environment while walking based on their route choice behavior and to identify factors that would stimulate pedestrians to select further routes to improve their walking activity. Specifically, the potential correlations between network features, trip characteristics, and personal factors were identified by analyzing the route choice characteristics. Next, the selected route was considered the preference for the environment while walking, and the influence of the street environment on the route choice behavior was analyzed. Finally, how the internal relationship between network features, trip characteristics, and personal factors affects the walking behavior of pedestrians was analyzed. The Chunliu Community, Dalian, was selected as the study site. It is an open and old community that was built in the 1990s. The site was selected as it has a dense road network and a diverse set of facilities for the pedestrians to select from.

## Literature

### Street Environment and Walking Behavior

According to the four-step model (which categorizes travel behavior into trip generation, trip distribution, mode choice, and trip assignment), the studies on the built environment may also be categorized into those based on walking generation (trip generation), the distribution of walking destination (trip distribution), the factors affecting walking frequency (mode choice), and the route choice. Most studies from the different fields of public health belong to the first three categories and have focused on measuring the overall walking volume, walking destination (walkability), and walking frequency to identify and analyze the environmental factors that promote an active and healthy lifestyle among residents (24). On the contrary, studies based on exploring the route choices are relatively scarce, even though the determinants of route choice may reveal important information regarding the role of the environment in influencing active travel behavior. In the field of urban planning and transportation, several studies were conducted to investigate different types of walking behaviors, such as the pedestrian crossing behavior at signalized and un-signalized intersections or in a street segment (25, 26); the patterns of crowd movement during an extreme event (27); and pedestrian volume/flow in different road segments (28). However, despite being focused on an important behavioral element, these studies could not capture pedestrians' experience of the environment during walking or their personal preferences for the street environment while walking.

On the other hand, route choice analysis would enable researchers to identify the street environment preferences of those who already walk, rather than those who are likely to walk, which would, in turn, form an important basis for developing urban planning and design policies for constructing pedestrian-friendly environments.

### Street Environment and Route Choice Behavior

The route choice process may conceptualize as pedestrians selecting a route with the highest utility among the various competitive choices of routes connecting the origin with the destination (29). Therefore, the route choice models are based on the hypothesis of utility maximization behavior (30). Different from the conventional approach to studying the street environment and walking behavior, route choice behavior analysis would enable a better understanding of pedestrians' preference for the street environment while walking by comparing the environmental characteristics of the selected route and the non-selected routes. Moreover, the process of street environment measurement comprises a series of linear segments, which facilitates the evaluation of the quality of the street environment and explains the impact of the micro-scale street environment attributes on the walking experience. Nonetheless, studies that have analyzed the correlation between the street environment and the walking behavior from the perspective of route choice behavior are scarce. This is because several of the features of the street environment, such as building design, signage, and streetscape, which may affect the experience and behavior of pedestrians, are difficult to quantify.

The collection and measurement of the data regarding these features are also difficult and time-consuming. Even if the data regarding pedestrian street networks are available, such data often have poor quality. Therefore, only a few studies have been conducted in this regard, with most of them having reported distance as the key determinant of route choice. For instance, the shortest route was revealed as the most preferred route among the competitive routes, leading to the inference that pedestrians frequently attempted to minimize distance (31, 32) and walking duration when selecting their routes, and there exists sufficient empirical evidence to demonstrate this (33, 34). The street environment was also reported to play a significant role in pedestrian route choice behavior, although only when the pedestrian's actual travel route deviated from the shortest distance route (35).

Although there have been great breakthroughs in the measurement of the street environment in the field of public health, such as audit tools enabling comprehensive and detailed measurements of the street environment at the micro-scale (36–40), analyzing the route choice behavior using these existing audit tools remains difficult. The existing audit tools typically comprise hundreds of environmental indicators, the collection of which is time-consuming, while the data would have little relevance for route choice analysis. Furthermore, there exist geographical discrepancies, which require extensive testing.

### Other Factors Affecting Route Choice Behavior

In addition to the street environmental features, pedestrians' route choices are also influenced by other factors, such as personal factors (gender and age), trip characteristics (trip purpose), and network features (41).

(1) Network features

Any type of walking requires pedestrians to use the existing spatial cognition records to connect the origin with the destination. Street network characteristics affect the cognitive effort required for pedestrians to navigate in an area when they move around in the urban environment (42). Extensive research has demonstrated the priority position of the route length factor or distance factor in pedestrians' route choice behavior. Therefore, it may be inferred that certain relationships exist between the route choice behavior and network topology as the distance is related to network topology (41). Accordingly, studies describing street networks should incorporate measures that capture a variety of configurational qualities, such as metrics and geometrics.

(2) Personal factors

While certain studies have proved that personal factors (such as gender and age) have little effect on route choice (43). Other studies have demonstrated that several differences exist in the route choices between different genders and ages, such as male individuals and the elderly prefer to select the shortest distance (43). Certain other studies have indicated that gender plays a role in the different perceptions of the environment, thereby affecting walking behavior. For instance, women were reportedly more inclined to select the environment with a high safety perception for walking activities (44). However, these studies did not explain why men and women reported different associations between walking and perceived environmental features, such as access to walking routes and destinations, pleasant scenery, traffic, and perceived safety (45–47).

(3) Trip characteristics

Studies on route choice have revealed that the synergistic effect of the street environment and route are likely to affect the route choice behavior. For instance, similar street environments reportedly lead to different route selection results in different areas (23). The expectations and the emerging evidence of the environmental influence on physical activity are likely to be context-specific (48). Therefore, it is reasonable to expect that route choice consideration may also vary according to the trip locations and objectives (23).

However, most of the previous studies have focused only on how the specific network characteristics, trip purpose, and personal factors influence the route choice behavior. For instance, the different area characteristics of the road network affect the spatial direction of route choice (42). In addition, different street environments along the way between different neighborhoods and destinations reportedly affect the route choice behavior as well (23), while the potential correlations between network characteristics, trip characteristics, and personal characteristics were not considered for the route choice characteristics.

### Study Design

This study focused on determining how the street environment influences walking preferences by analyzing the route choice behavior. While the area-based studies measure the built environment and the overall walking level (walking frequency and duration) in a particular area, this study involved measuring the linear-segment street environment, which could better capture the walking experience. The various aspects of walking behavior were measured using the disaggregated method, which focuses on the route choice behavior level among all the characteristics of walking behavior and enables identifying the street environment-related factors that lead to increased walking distance during utilitarian walking as well as increased levels of physical activity.

However, studies analyzing the correlation between the street environment and walking behavior from the perspective of route choice behavior are limited as it is difficult to quantify the street environment. Although great breakthroughs have occurred in the quantification of the street environment in the field of public health, certain limitations remain, such as regional variability and the time-consuming nature of data collection. The limited literature that is available in this regard reports that the street environment plays a significant role in the route choice behavior only when the actual travel route undertaken by the pedestrian deviates from the shortest distance route (35).

In addition to the street environment features, pedestrians' route choices are also influenced by other factors, such as personal factors, trip characteristics, and network features, which might interfere with the pedestrians' route choice and individual differences regarding the environmental preferences. Therefore, determining which of these factors are interconnected and influencing pedestrian behavior is critical.

In this context, this study focused mainly on analyzing the pedestrians' route choice characteristics and identifying the street environment-related factors affecting the route choice based on the probability of pedestrians' non-selected shortest distance route. Meanwhile, the discrepancies arising from the street environment, network features, gender, trip characteristics, and route choice characteristics were determined, and potential correlations between these factors were identified.

## Methods

### Study Site

Since the measurement of route choice behavior is hindered by difficulty related to data collection and the huge workload of street environment audits, the scope of activities in the present study was limited to reduce the cost of data collection. In order to better analyze the route choice behavior, an area with a dense road network and diverse destination facilities was selected as the study site so that a higher number of possible routes are available for selection in the process of walking to facilitate behavior analysis.

**Figure 1:**
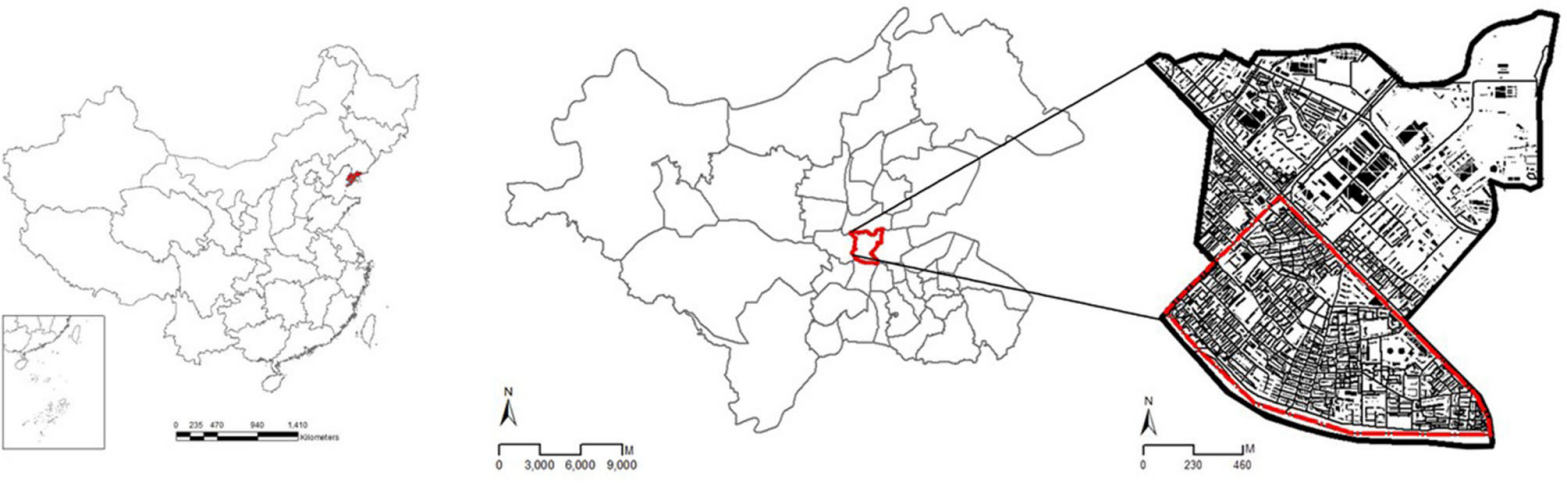
Study site.

Accordingly, the Chunliu community in Dalian, China (Figure 1), was selected. This is an open and old residential area that was constructed in the 1990s. The walking behavior of the residents, including route choice, must have become a habit integrated into their daily lives as most of the residents of any area are familiar with the residential environment.

### Data Collection

This study was different from the area-based environment and behavior research in terms of encountering difficulty in the detailed data collection on the route undertaken by the pedestrians. Although several methods were available to resolve this issue, no single evaluation method could satisfy all requirements as different methods were suitable for different types of environmental attributes (49). Therefore, to record the pedestrian route behavior in Chunliu, an unobtrusive tracking method that did not rely on the subjects' memories and could record the route reliably was employed. In particular, we invited participants to install the “2bulu” app, which is a piece of software that records behavioral trajectories. We then encouraged participants to send us the routes they took to reach their first destination from their residence after having completed their trips. The route data were collected between 3:00 pm and 5:00 pm every Sunday for the whole month of October 2020, including the routes that the adults undertook to their first destination from their respective residential locations. The privacy concerns were addressed through an agreement from the respondents after the completion of the observations. The final data collected included the route data from 219 residents.

### Generation of the Street Environment

The street environment factors collected from the environmental audit tools and SWATCH tools (50), the only audit tools available for the route choice analysis, were reorganized and incorporated into the local street environment. Subsequently, the physical aspects were classified according to its functional characteristics into roadway features, streetscape, pedestrian infrastructure, and facilities (Table 1).

**Table 1:** Categories and description of the street environment tool used to collect data.

**Street environmental factors**	**Categories**	**Description**	**Measuring scales**
Roadway features	Sidewalk width	The actual width of the pedestrian pavement	Continuous scale
	On-street parking	Parking lot	1
		Road parking	2
		Sidewalk parking	3
	Sidewalk walkability	Poor=sidewalk is extremely difficult or nearly impossible to go across	1
		Fair=side walk has some unevenness or obstacles, but it can still be navigated	2
		Good=sidewalk is in pristine or near pristine condition, very easy to go across	3
	Traffic control signal at intersections	The number of signal which at an intersection have a pedestrian walk signal, stop light or stop sign	Continuous scale
	Driveway width	The actual width of the Driveways	Continuous scale
Streetscape	DH	The width of the street divided by the height of the building	Continuous scale
	Green spaces	Poor=Some tree ponds only	1
		Fair=Some tree ponds and parterre	2
		Good=both tree ponds, parterre and green spaces	3
	Characteristics of street walls	No street walls	0
		The wall material is railing	1
		The wall material is solid wall	2
Pedestrian infrastructure	Garbage bins	The number of garbage bins	Continuous scale
	Streetlights	The number of street lights or lampposts	Continuous scale
	Benches	The number of seats along the street in which people can take a break	Continuous scale
Facilities	Shops	The proportion of the street front occupied by grocery, shop	Continuous scale
	leisure facilities	The proportion of the street front occupied by restaurants, banks, parks, entertainment	Continuous scale
	Bus stops	The proportion of the street front occupied by bus stops	Continuous scale

The basic data at the macro-level, such as route networks and buildings (contours, layers, etc.), were collected from the OSM map. The points of interest data related to the facilities, such as retail establishments, catering services, public services, parking lots, medical treatment, and bus stations, were extracted from the Baidu Map. The quality and quantity of the street environment features, such as width and walkability of the sidewalk and driveways and building height, were ensured by recruiting an auditor well-trained in performing the survey and collecting data for each road segment along the route. The variables that concerned the functional condition of the pedestrian infrastructure (on-street parking, sidewalk walkability, green spaces, and characteristics of street walls) and specific values (sidewalk and driveway width, DH) were expressed in length-weighted average values.

### Analytical Method

In order to understand the pedestrians' preference for the environment while walking, the characteristics of the route choice behavior of pedestrians were analyzed.

First, the actual routes were categorized into selected and non-selected shortest distance routes. Next, the differences between the selected and non-selected shortest distance routes, in terms of network features, gender, and trip characteristics, were determined to identify the potential factors.

Specifically, the walking distance, the number of intersections, and the detour ratios were the network features determined for revealing the route choice characteristics. The detour ratio was defined as the route directness, which is often associated with active travel and is expressed as follows:


$$\begin{array}{l} RDI=\frac{E}{R} \end{array}$$

Here, *E* denotes the Euclidean between the origin and the destination, and R denotes the route distance. The RDI value quantifies the route deviation from a Euclidean benchmark. The closer the index value (0 ≤ RDI ≤ 1) is to 1, the more direct the route is the indicated route.

In addition, the trip purposes defined were shopping, life-service, leisure, and transportation which were selected as these were the frequently visited destinations during utilitarian walking. Moreover, we collected the route data from randomly selected the pedestrians at their residence, and these pedestrians included those who walk alone or with an accompanist. It was considered that different genders perceived the street environment differently and that this difference could affect the walking behavior. Also, according to the travel status, we added the factors for the pedestrians who walk alone or with an accompanist.

Finally, the binary logit regression model was utilized to identify the street environment factors that influenced the pedestrians to select or not select the shortest distance. The model was divided into two parts: The first part was the basic model, which simply analyzed the influence of the street environment on walking preferences, and the second part included the trip characteristic factors (trip purpose and travel status) and the interaction between these factors into the model to explore the influence of the street environment on the route choice behavior.

All analyses were conducted using SPSS 23.0.

## Results

### Analysis of the Route Choice Characteristics

(1) Network features

In order to understand the differences in the network features between the selected and non-selected shortest distance routes, the mean differences in route distance, directness, and the number of intersections crossed were estimated, and a one-way ANOVA was conducted for each of these attributes to evaluate whether the differences were statistically significant.

Table 2 presents the results of one-way ANOVA conducted between the selected and non-selected shortest distance routes. The one-way ANOVA results indicated the significant differences at the 5% level in the network features between the selected and non-selected shortest distance routes within the pooled sample. It was revealed that the non-selected shortest distance route had a typically longer distance (*F* = 38.019, *P* = 0.000), smaller route directness (*F* = 28.887, *P* = 0.000), and less intersections crossed (*F* = 7.568, *P* = 0.006) than the selected shortest distance route. These significant differences showed that route distance is the key factor in the differences between the selected shortest route and the non-selected shortest route. As the route distance increases, the number of intersections traversed increases. The difference in route directness also shows that their spatial cognitive ability will decrease when pedestrians walk longer.

**Table 2:** Differences in network features between selected and non-selected shortest routes.

**Walking preferences**	**Route distance**	**Route directness**	**No. of intersections crossed**	***N***
selected shortest routes	278.809	0.797313	13.82192	145
non-selected shortest routes	460.7973	0.700779	9.108108	74
F	38.019	28.887	7.568	
P	0.000***	0.000***	0.006***	

*P < 0.1, **P < 0.05, ***P < 0.01.

(2) Gender

The chi-squared test was conducted to explain the difference in the walking preferences and gender. However, the results revealed no significant differences between the gender in terms of the selected and non-selected shortest distance routes, which was consistent with the findings reported in previous studies (43). It shows that the perceived route choice of walking is not necessarily related to the intention to the personal factors (gender) alone, but the combination of multiple environmental factors (51). It also proved that the issues had been partially addressed among transportation and urban planning researchers in the discussion of whether travel is derived. Derived models suggest that travel is engaged in only as a means to an end destination, and thus, the travel behavior itself is largely, if not completely, influenced by extrinsic utility variables (such as cost, distance to destination, and transportation infrastructure) (10).

Next, the differences between network features and gender were analyzed to explain the potential relationship between the two. The results of the one-way ANOVA between gender and the route distance, directness, and the number of intersections revealed no significant differences between the gender and route distance. We can attribute the peculiarity of our analytical results to the specific characteristics of the utilitarian walking, which generally involves shorter and faster trips than recreational walking. The shorter and faster trips lead to the difference in the cognition of walking distance. It means that there are no differences between gender in the cognition of walking distance during utilitarian walking (short-distance travel).

(3) Trip characteristics

The results of the chi-squared test conducted between the different proportions of trip purposes, including shopping, living service, leisure, and transportation, for the walking preferences (Table 3) revealed no difference between the proportion of trip purposes in terms of the selected or non-selected shortest distance route (χ^2^_(3)_ = 2.854, *P* = 0.415).

**Table 3:** Chi-square test between trip purpose and walking preferences, gender, and travel status.

**Trip purpose**	**Walking preferences**		**Gender**		**Travel status**	
	**Non-selected shortest**	**Selected shortest**	**Male**	**Female**	**Walk alone**	**With accompanist**
Shopping	32(39.5%)	49(60.5%)	33(47.1%)	37(52.9%)	70(86.4%)	11(13.6%)
Life-service	19(26.8%)	52(73.2%)	34(54%)	29(46%)	63(88.7%)	8(11.3%)
Leisure	6(37.5%)	10(62.5%)	2(18.2%)	9(81.8%)	11(68.8%)	5(31.3%)
Transportation	17(33.3%)	34(66.7%)	26(66.7%)	13(33.3%)	39(76.5%)	12(23.5%)
χ^2^	2.854		9.594		6.286	
*p*	0.415		0.027**		0.098*	

*P < 0.1, **P < 0.05, ***P < 0.01.

Furthermore, one-way ANOVA was performed between the proportion of trip purpose and network features (Table 4), which revealed no difference between the two, except for the route distance factor at the 10% statistically significant level. A chi-squared test was then conducted between the proportion of trip purpose and gender (Table 3), the results of which revealed a significant difference at the 5% level between the two (χ^2^_(3)_ = 9.594, *P* = 0.027), indicating that there is no difference between gender and trip purpose. It also indicated that people of different genders might have different preferences for trip purposes. It suggests that individuals have a varying degree of motivation and utility for specific destinations (52).

**Table 4:** One-way ANOVA between trip purpose and network features.

**Trip purpose**	**Route distance**	**Route directness**	**No. of intersections crossed**
Shopping	318.3102	0.760904	7.807229
Life-service	314.3331	0.757494	7.652174
Leisure	338.5463	0.735418	7.6875
Transportation	411.7811	0.789789	7.529412
*F*	2.432	0.945	0.027
*P*	0.066*	0.42	0.994

*P < 0.1, **P < 0.05, ***P < 0.01.

Moreover, the results of the chi-square test conducted between the travel status and trip purpose revealed that there was a difference between the pedestrians who walked with an accompanist and the choice of trip purpose (χ^2^_(3)_ = 6.286, *P* = 0.098) (Table 3). Then, one-way ANOVA was performed between travel status and network features (Table 5) which showed that there was a difference at the 5% significant level between travel status (whether pedestrians walked with an accompanist or not) and network features (route distance and number of intersection). It means that when pedestrians walk with an accompanist, they will be walking longer and crossed more intersections than a pedestrian walking alone. It also means that the travel status can affect the difference in cognition of walking distance, which leads to differences in walking preferences for route choice.

**Table 5:** One-way ANOVA between travel status and network features.

**Travel status**	**Route distance**	**Route directness**	**No. of intersections crossed**
Walk alone	319.9093783	0.760439339	7.349726776
with accompanist	441.1648935	0.786322218	9.388888889
*F*	9.256	1.131	4.118
*P*	0.03**	0.289	0.044**

*P < 0.1, **P < 0.05, ***P < 0.01.

Overall, there is a correlation between gender, trip purpose, and travel status. However, there are also differences between trip characteristics (trip purpose and travel status) and network features. Also, the same differences existed between network features and walking preferences (the selected and non-selected shortest distance routes). Therefore, it was inferred that the interaction between trip purpose and travel status could affect route choice behavior.

### Influence of Street Environment on Route Choice Behavior

The results of the binary logistic regression model, which explained the influence of the street environment on route choice behavior, are presented in Table 6. The on-street parking (*p* < 0.05), the number of garbage bins (*p* < 0.01), and streetlights (*p* < 0.05) were observed to impact the pedestrians' selection of the shortest distance routes, indicating that pedestrians are likely to walk to greater distances between their origin and destination. Specifically, the number of garbage bins and streetlights in the street environment were observed to have a positive effect on the pedestrians' selection of the shortest distance route and were, therefore, inferred as important factors stimulating longer distance walks. This could be because the presence of garbage bins and streetlights along the route reduced the fear of crime in the mind of pedestrians and improved the chances of personal safety while walking. The garbage bins are usually placed on both sides of residential buildings for the elimination of domestic garbage. The fact that pedestrians are more likely to select these routes indicates that the routes of garbage bins bring convenience to pedestrians. In addition, several studies have reported that maintaining street cleanliness and placing streetlights improves street safety (53–55).

**Table 6:** Street environmental factors affecting non-selected shortest routes.

**categories**	***B***	**Sig**.
Roadway features
Sidewalk width	0.024	0.782
On-street parking	−0.683	0.012**
Sidewalk walkability	0.119	0.686
Traffic control signal at intersections	−0.018	0.81
Driveways width	0.034	0.601
Streetscape
DH	−0.494	0.194
Green spaces	−0.205	0.56
Characteristics of street walls	−0.031	0.949
Pedestrian infrastructure
Garbage bins	0.292	0.005***
Benches	0.182	0.191
Streetlights	0.095	0.042**
Facilities
Shops	1.245	0.339
Leisure facilities	0.41	0.746
Bus stops	−3.882	0.476
Constant	−0.795	0.545

*Hosmer and Lemeshow test 0.37*.

*P < 0.1, **P < 0.05, ***P < 0.01.

Sidewalk parking was also more likely to prevent pedestrians from selecting the shortest distance route, which is contrary to what is commonly expected. Generally, it is assumed that sidewalk parking would be a hindrance to walking, thereby reducing the walking quality of pedestrians. The peculiar analytical results obtained regarding this in this study could be attributed to the fact that pedestrians prefer to walk on trails due to the inadequate provision of good quality walking conditions and the lack of policies that satisfy the demands of pedestrians. In the study site of this study, most trails had been occupied for parking cars, due to which the pedestrians had to walk on the driveway.

Table 7 presents the results of the models for the effect of street environment on route choice behavior when including travel status, trip purpose, and the interaction between these factors. As in previous analysis, trip purpose was observed to have no effect on the pedestrians' selection or non-selection of the shortest distance route. However, in the interaction between travel status and trip purpose in the same street environment, pedestrians walking for leisure purposes with an accompanist were positively influenced for selecting the shortest distances compared to when walking for other purposes while walking alone (*p* < 0.1). This implied that the pedestrians with accompanists could not experience the impact of the street environment while walking for leisure purposes, such as parks, because of which they were more willing to select the shortest distance route and reduce their walking distance along the way.

**Table 7:** Other factors affecting non-selected shortest routes.

**Street environmental factors**	***B***	**Sig**.	***B***	**Sig**.	***B***	**Sig**.
**Roadway features**
Sidewalk width	0.024	0.786	0.034	0.693	0.014	0.874
On-street parking	−0.683	0.013**	−0.724	0.01**	−0.703	0.012**
Sidewalk walkability	0.12	0.686	0.145	0.633	0.211	0.493
Traffic control signal at intersections	−0.018	0.808	0.008	0.916	−0.004	0.955
Driveways width	0.034	0.602	0.022	0.709	0.035	0.582
**street scape**
DH	−0.495	0.194	−0.602	0.117	−0.567	0.141
Green spaces	−0.203	0.566	−0.136	0.701	−0.126	0.726
Characteristics of street walls	−0.03	0.95	−0.034	0.944	0.041	0.932
**Pedestrian infrastructure**
Garbage bins	0.293	0.005***	0.31	0.003***	0.324	0.002***
Benches	0.183	0.193	0.175	0.21	0.183	0.189
Streetlights	0.095	0.045**	0.085	0.07*	0.083	0.083*
**Facilities**
Shops	1.243	0.361	1.206	0.375	1.168	0.392
Leisure facilities	0.409	0.747	0.227	0.857	0.171	0.893
Bus stops	−3.891	0.476	−3.918	0.486	−4.315	0.451
**Other factors**
with accompanist	−0.013	0.977				
Shopping				0.349		
life-service			−0.115	0.795		
Leisure			−0.755	0.114		
Transport			−0.275	0.689		
Walk alone*shopping						0.345
With accompanist*life-service					−0.369	0.362
With accompanist*leisure					−0.801	0.07*
With accompanist*transport					−0.244	0.748
		0.562	−0.251	0.854	−0.312	0.816

*Hosmer and Lemeshow test 0.369*.

*Hosmer and Lemeshow test 0.727*.

*Hosmer and Lemeshow test 0.805*.

*P < 0.1, **P < 0.05, ***P < 0.01.

## Discussion and Conclusion

This study focused on improving the street environment to increase the walking distance during utilitarian walking for reducing the health issues arising due to sedentary lifestyles. An unobtrusive tracking method was adopted to observe the route choice characteristics, and the relationship among the street environment, trip purpose, travel status, and walking behavior was analyzed.

The results revealed that the pedestrians' selection or non-selection of the shortest distance route was affected by the network features named “route”. In particular, the route distance factor was observed to play a significant role, which was consistent with the conclusions of most of the previous studies. Moreover, the route detour ratio (RDI) reflecting the route directness was analyzed. This RDI is often associated with active travel, with a higher detour ratio implying a lower cognitive ability of space in pedestrians. This spatial cognitive ability may be influenced by location cognition (OD position) and metric cognition (distance). However, in addition to the discipline of geography, the directional distance in the discipline of topology also affects the spatial cognition (42) as distance also has a certain relationship with network topology (41).

While the factors gender, trip purpose, and travel status are not expected to directly affect route choice, potential correlations were observed in network features between different trip characteristics (trip purpose and travel status). This also demonstrated the complexity of the studies conducted on route choice as there are several latent variables to consider and control, which might also be involved and be acting synergistically.

Furthermore, certain pedestrian infrastructure features, such as garbage bins and streetlights, were observed to have a positive safety perception and thereby increase the walking distance. This result could have been obtained because of certain latent variables being ignored, which could have affected the route choice, such as the differences in the subjective perception of pedestrians. This result, therefore, encourages studying the effect of the street environment on the subjective perception of pedestrians as an interesting topic in terms of pedestrian safety and convenience. From the route choice characteristic aspects, there are some regularities in route choice, such as pedestrians preferring to choose the roads with relatively concentrated traffic flow. It may be due to these roads have high accessibility to any other destination. In addition, the deviation from the shortest distance occurred in the area which was near to the pedestrians' residences and pedestrians were generally willing to choose the trails and roads inside the residential area, because these routes would reduce the interference of traffic flows.

The results of this study also revealed that pedestrians are more likely to select the shortest distance route when they are walking for leisure activities along with an accompanist. This result led to the inference that the pedestrians are not willing to change their habit of always selecting the shortest distance route and increase their walking distance when walking along with an accompanist. This inference raises interesting concerns regarding the street environment policy for this study site. Accordingly, it becomes important to consider improving the shortest distance routes connecting the residences with the destinations of leisure activities through a better pedestrian infrastructure, such as providing a cleaner and lighter environment, to increase the perception of safety. This would also improve the physical activity of pedestrians by increasing their walking frequency to the destinations of leisure activities and thereby increasing participation in such activities.

Certain results of this study were also contrary to those reported in previous studies, such as the results obtained in regard to the on-street parking. The contradictory results obtained for this factor demonstrate the difficulty in collecting and interpreting the street environment data at the microscale due to the high number of potential variables to consider and the high number of potential correlations among these variables (56). Another possible reason for this contradictory result could be certain variables that were missing from the present analysis. For instance, sidewalk parking was more common in the study site, especially on trails, and walking on trails reduced motor vehicle intervention, which could have served as a factor. Similarly, a few important street environment variables were not captured in this study audit such as traffic flow. The findings merit the consideration of interventions on trails, such as establishing a parking lot to resolve the sidewalk parking issue. Therefore, ensuring a safe street environment and improving the connectivity between the origins and destinations could significantly improve the quality of utilitarian walking (57).

According to the results obtained in this study and the aforementioned discussion, the following conclusions were drawn:

(1) Distance is a determinant of route choice behavior. Moreover, trip purpose and travel status were also potential factors influencing pedestrians' route choice, and there was interactional correlation among these factors.
(2) The street environment would affect pedestrians' walking preference for the environment. Specifically, on-street parking, garbage bins, and streetlights could influence pedestrians to select longer distance routes.
(3) In the street environment, pedestrians with an accompanist walking for leisure purposes were positively influenced for selecting the shortest distances compared to when walking alone for other purposes in other genders.

In addition to the substantive findings stated previously, this study also contributed to determining the relationship between the street environment and walking behavior. The results from the present analysis of the micro-level street environment suggested that route features and pedestrian infrastructure are likely to increase the walking distance (although the additional distance is usually limited) by improving the walking quality of pedestrians. These findings were consistent with those of the previous studies reported on the relationship between the macro-level built environment and walking behavior, such as the report stating that pedestrians were less responsive to environmental quality than to recreational walking (2).

However, the relatively small sample size of this study allowed only the evaluation of the existing street environment and contrasting and complementing the typical results of the correlation studies at the micro-scale. Moreover, since this study was limited to just one community to reduce the data collection costs, caution should be maintained when generalizing the obtained results. Therefore, further research is warranted to develop comprehensive, simple, and low-cost environment audit tools for the measurement and basic analysis of the street environment data. In addition, a general and predictable route choice model should be built to serve as a support tool. For example, we only considered pedestrians' first destination from their respective residential locations and didn't consider the influences of pedestrians' overall travel plan that may be the other influencing factors affecting our results. In future, the number of studies conducted on how pedestrians experience the street environment to form environmental preferences would increase, which would provide a further reliable foundation for studying the relationship between the street environment and walking behavior.

This study also has practical significance as it could assist in policymaking and prioritization of projects aimed at assessing and improving the street environment in settlements. In addition, this study provides clues for constructing pedestrian-friendly settlements to increase the utilitarian walking distances.

## Data Availability Statement

The original contributions presented in the study are included in the article/supplementary material, further inquiries can be directed to the corresponding author/s.

## Author Contributions

LJ contributed to the conceptualization, methodology, investigation, writing—original draft, and visualization of the study. WL performed the supervision and project administration. PS performed the writing—review and editing. All authors contributed to manuscript revision, read, and approved the submitted version.

## Funding

This study was supported by the Fundamental Research Funds for the Central Universities with funding number: DUT20RC (3) 051.

## Conflict of Interest

The authors declare that the research was conducted in the absence of any commercial or financial relationships that could be construed as a potential conflict of interest.

## Publisher's Note

All claims expressed in this article are solely those of the authors and do not necessarily represent those of their affiliated organizations, or those of the publisher, the editors and the reviewers. Any product that may be evaluated in this article, or claim that may be made by its manufacturer, is not guaranteed or endorsed by the publisher.
